# Therapeutic outcome and related predictors of stereotactic body radiotherapy for small liver-confined HCC: a systematic review and meta-analysis of observational studies

**DOI:** 10.1186/s13014-021-01761-1

**Published:** 2021-04-08

**Authors:** Yanyan Long, Yan Liang, Shujie Li, Jing Guo, Ying Wang, Yan Luo, Yongzhong Wu

**Affiliations:** 1 Present Address: Department of Radiation Oncology Center, Chongqing University Cancer Hospital, Chongqing Cancer Institute, Chongqing Cancer Hospital, No. 181 Hanyu Road, Shapingba District, Chongqing, 400030 China; 2 Chongqing Key Laboratory of Translational Research for Cancer Metastasis and Individualized Treatment, Chongqing University Cancer Hospital, Chongqing Cancer Institute, Chongqing Cancer Hospital, Chongqing, 400030 China; 3 Division of Biological Science, University of California San Diego, San Diego, CA 92122 USA

**Keywords:** Hepatocellular carcinoma (HCC), Stereotactic body radiotherapy (SBRT), Meta-analysis, Prognosis

## Abstract

**Background and purpose:**

Stereotactic body radiotherapy (SBRT) is a promising ablative modality for hepatocellular carcinoma (HCC) especially for those with small-sized or early-stage tumors. This study aimed to synthesize available data to evaluate efficacy and explore related predictors of SBRT for small liver-confined HCC (≤ 3 lesions with longest diameter ≤ 6 cm).

**Materials and methods:**

A systematic search were performed of the PubMed and Cochrane Library databases. Primary endpoints were overall survival (OS) and local control (LC) of small HCC treated with SBRT, meanwhile, to evaluate clinical parameters associated with treatment outcome by two methods including subgroup comparisons and pooled HR meta-analysis. The secondary endpoint was treatment toxicity.

**Results:**

After a comprehensive database review, 14 observational studies with 1238 HCC patients received SBRT were included. Pooled 1-year and 3-year OS rates were 93.0% (95% confidence interval [CI] 88.0–96.0%) and 72.0% (95% CI 62.0–79.0%), respectively. Pooled 1-year and 3-year LC rates were 96.0% (95% CI 91.0–98.0%) and 91.0% (95% CI 85.0–95.0%), respectively. Subgroup comparisons regarding Child–Pugh class (stratified by CP-A percentage 100%, 75–100%, 50–75%) showed there were statistically significant differences for both 1-year and 3-year OS rate (p < 0.01), while that regarding number of lesions, pretreatment situation, age (median/mean age of 65), macrovascular invasion, tumor size, and radiation dose (median BED_10_ of 100 Gy), there were no differences. In subgroup comparisons for LC rate, it showed number of lesions (1 lesion vs. 2–3 lesions) was significantly associated with 1-year LC rate (p = 0.04), though not associated with 3-year LC rate (p = 0.72). In subgroup comparisons categorized by other factors including pretreatment situation, age, CP-A percentage, macrovascular invasion, tumor size, and radiation dose, there were no significant differences for 1- or 3-year LC rate. To further explore the association between CP class and OS, the second method was applied by combining HR and 95% CIs. Results indicated CP-A was predictive of better OS (p = 0.001) with pooled HR 0.31 (95% CIs 0.11–0.88), which was consistent with subgroup comparison results. Concerning adverse effect of SBRT, pooled rates of grade ≥ 3 hepatic complications and RILD were 4.0% (95% CI 2.0–8.0%) and 14.7% (95% CI 7.4–24.7%), respectively.

**Conclusion:**

The study showed that SBRT was a potent local treatment for small liver-confined HCC conferring excellent OS and LC persisting up to 3 years, even though parts of included patients were pretreated or with macrovascular invasion. CP-A class was a significant predictor of optimal OS, while number of lesions might affect short term tumor control (1-year LC). Tumor size and radiation dose were not vital factors impacting treatment outcome for such small-sized HCC patients. Because of the low quality of observational studies and heterogeneous groups of patients treated with SBRT, further clinical trials should be prospectively investigated in large sample sizes.

**Supplementary Information:**

The online version contains supplementary material available at 10.1186/s13014-021-01761-1.

## Introduction

Hepatocellular carcinoma (HCC), the most common primary liver malignancy, is regarded as the sixth most commonly diagnosed solid tumor, and the second leading cause of cancer-related death worldwide [1]. The prognosis and treatment options depend not only on the tumor stage but also on liver function and general condition of patients [2]. Liver transplantation, surgical resection, and local ablative therapies are applied with curative intent for patients with small early-stage HCC. However, liver transplantation is limited by organ availability and strict candidate selection criteria [3]. Surgical resection is commonly contraindicated due to presence of portal hypertension, cirrhotic liver with poor liver function or other medical comorbidities [4]. Thus, in a large proportion of early-stage patients, local ablative therapies are the mainstay of treatment. These include radiofrequency ablation (RFA), microwave ablation (MWA), percutaneous ethanol injection (PEI), and external radiation therapy. But some tumors are not suitable for RFA or MWA as anatomical difficulties in approaching some lesions, such as those adjacent to major vessels, biliary trees, diaphragms, or heat sink effect of RFA [5]. And PEI is associated with incomplete necrosis in most HCCs > 2 cm and suffers a high local recurrence rate with 49% in lesions exceeding 2 cm [6]. In addition, the distribution of alcohol inside the lesion cannot be well governed and usually does not extend beyond the cirrhotic fibrous tissue surrounding the tumor.

Classically, radiation therapy directed at the liver was of limited use, due to radiation-induced liver disease (RILD) [7]. However, along with the development of new delivery techniques, as well as new radiotherapeutic modalities, this has changed. Stereotactic body radiation (SBRT) has been pioneered by several centers worldwide as an alternative local ablative therapy for early small HCC [8–12]. For those tumors, SBRT precisely delivers high doses of radiation in just a few fractions conforming to the target volume with a low risk of radiation injury. The Asia-Pacific Primary Liver Cancer Expert meeting (APPLE), an association of liver cancer experts in the Asia-Pacific region, recommended application of SBRT for early-stage or small-sized HCC [13], especially if surgical resection or percutaneous ablative therapies are difficult, unfeasible, or rejected. This approach is also used as a salvage treatment for tumor recurrence after local radical therapies or for residual cancer after surgical resection or percutaneous ablative attempts. Nevertheless, most of the evidence based on observational studies. The role of SBRT in small HCC have not been well established due to the lack of high-level evidence. Correspondingly, it will lead to a lack of recognized predictors of treatment outcome (such as OS and LC) which are extremely important for optimal treatment planning.

Although randomized controlled trials provide the strongest evidence, they are time-consuming and labor-consuming. Clinical practices are often based on multiple smaller trials or clinical observations as well. Therefore, a meta-analysis of observational studies might be one of the best available options to evaluate the feasibility and efficacy of treatment and to provide useful information for clinical decision-making [14]. The aim of our study is to perform the first systematic review and meta-analysis of patient-specific outcomes of SBRT for small liver-confined HCC (≤ 3 lesions with longest diameter ≤ 6 cm) from a series of observational studies, and meanwhile, to comprehensively explore potential factors that can help clinicians in the therapeutic choice, determine stratification factors for future studies in this subset of patients.

## Materials and methods

### Study protocol

This study adhered to the Preferred Reporting Items for Systematic Reviews and Meta-Analysis (PRIMSA) guidelines. A systematic electronic search of PubMed and Cochrane Library databases was conducted on April 12, 2020, and re-run on April 30, 2020. We used the following search query: “(stereotactic body radiotherapy OR stereotactic ablative radiotherapy OR Cyber*Knife OR Gamma*Knife OR SBRT OR SABR) AND ( adenoma, hepatocellular OR Hepatocellular Carcinoma OR hepatic malignancy OR liver cancer OR hepatic neoplasm OR liver neoplasm)” to identify studies on SBRT for HCC patients published from 2000/01/01 to 2020/04/30 in English. Unpublished or other language studies were not included in the search. Detailed search query was shown in Additional file 2: Supplementary Data 1-Part 1. The search terms were designed to find studies using SBRT or stereotactic ablative radiotherapy (SABR) to treat HCC, emphasizing clinical outcomes or adverse effect rather than technical perspectives.

### Selection criteria and data extraction

After initial searching, it returned 444 results in total (346 in PubMed and 98 in Cochrane). Then studies were filtered to exclude duplicated studies, conference abstracts, reviews, letters, editorials, case reports, lab studies, and studies with irrelevant subjects using titles and citation. The remaining studies were reviewed by firstly reading abstract or patients character table, and/or next step comprehensively reading the full text to determine whether they fully met the inclusion criteria. The following inclusion criteria were used: (1) prospective and retrospective studies, reporting results of SBRT on small liver-confined HCC (1–3 lesions in liver with maximum single tumor diameter ≤ 6 cm, no lymph node or extrahepatic metastasis), (2) provision of treatment outcome (OS or LC) or adverse effect; (3) inclusion of over 10 patients with HCC treated with SBRT; and (4) SBRT performed in < 10 fractions. In cases of multiple studies from one institution with overlapped patients, the following criteria were used, prioritized in numerical order, to determine inclusion: (1) study with the largest number of patients; (2) most recently published study. As this study involves different endpoints and different statistical methods to synthesize, we will properly sort the studies from same institution to different category according to the content of the paper. All in all, the aim is to calculate the same index without using repeated patients. Exclusion criteria were as follows: (1) unable to obtain full text; (2) SBRT was exclusively used as a bridge to liver transplantation; (3) combined with other anti-tumor treatment simultaneously (RFA, TACE, targeted therapy, immunotherapy, chemotherapy, et al.), but sequential therapy is allowed as long as there is at least 1-month interval; and (4) hypo-fractionated radiotherapy. All procedures to identify eligible studies were performed by two independent researchers (YYL and SJL). Any disagreement was resolved by discussion and mutual consent of the above two researchers and another researcher (YW).

The following data were obtained from original articles: (1) general information including authors, publication year, time of study, study design, country, number of patients and lesions, pre-treated or not, sex, and age; (2) clinical information including Child–Pugh class, ECOG (Eastern Cooperative Oncology Group) performance status, viral etiology, tumor vascular invasion, and tumor size; (3) treatment information and outcomes including SBRT dose, fractionation scheme, BED (Biologically Effective Dose), OS rate, LC rate, grade ≥ 3 hepatic complication, and RILD; (4) predictors for OS or LC and related HR with 95% CIs if studies supplied or could be calculated from available numerical data using methods reported by Tierney et al. [15].

### Study definitions

There, small liver-confined HCC was defined as 1–3 lesions in liver with maximum single tumor diameter ≤ 6 cm, no lymph node or extrahepatic metastasis. We included one study [16] with tumor volume ≤ 100 cc as diameter is calculated from volume, assuming tumor is spherical, which satisfied the criteria.

Treatment response was assessed using the Response Evaluation Criteria in Solid Tumors (RECIST) or modified RECIST (mRECIST) criteria on multiphase CT or MRI images performed after treatment, with very few unavailable [11, 12, 17, 18]. Local control (LC) was defined as absence of progression for target lesion (PTV). Overall survival (OS) and LC were estimated starting from the date of SBRT to the date of death or the final follow-up, and to the date of treated tumor progression or last follow-up, respectively, using the Kaplan–Meier method.

SBRT induced hepatic toxicity classification was according to Common Terminology Criteria for Adverse Events (CTCAE) for most studies, and very few based on Toxicity criteria of the Radiation Therapy Oncology Group (RTOG) and the European organization for research and treatment of cancer (EORTC) [10].

The definition of RILD was slightly different in different studies [10, 17–19], of which there are two types: classic RILD and non-classic RILD. Classic RILD was defined as anicteric hepatomegaly and ascites, or elevation of alkaline phosphatase more than twice above the upper limit of normal or baseline level, and non-classic RILD was defined as an elevation in the level of transaminases or bilirubin, which was graded according to CTCAE, or a decline in liver function measured by a worsening of CPS ≥ 2 points.

### Quality assessment

Because most (12/14) of the included studies were retrospective, the Newcastle–Ottawa Scale (NOS) [40] was applied to assess the quality of included studies by two investigators independently (SJL and JG). Studies with NOS scores of 7–9 were regarded as high-quality studies, and those with scores of 4–6 were considered medium-quality studies.

### Data synthesis and statistical methods

The pooled estimated 1-year OS rate, 3-year OS rate, 1-year LC rate, 3-year LC rate, grade ≥ 3 hepatic complication rate, and RILD rate were derived. Meta and Metafor Packages in R software were utilized to accomplish meta proportion analysis [20]. Raw proportion and other four methods (PLN, PLOGIT, PAS, PFT) to transform of raw proportion were performed for further analyses. By normal distribution test (Additional file 2: Supplementary Data 1-Part 3) and carefully comparing the five methods, we chose the best Logit transformation of raw proportion (PLOGIT) for final analyses to increase validity. The PLOGIT is calculated as the log of raw proportion divided by one minus the raw proportion. logit (p) = log (p/(1 − p)).

The prognostic values of common clinical factors for treatment outcome (OS and LC) were explored through two different ways: (1) by subgroup comparisons, categorized by potential predictive factors using R meta proportion subgroup analysis (which is a method to explore heterogeneities originally); (2) by combining HR and 95% CIs when there were at least three studies concerning same factor. If there were only two studies for certain predictor, we will not apply this method in order to avoid bias. The analyses were carried out using STATA software “metan” order. Multivariate data were preferable to univariate data if both were presented. However, univariate data were acceptable if multivariate results were not available. The pooled HRs were shown in the form of a forest plot. HR > 1 indicated poor survival or local control response when referring certain index, and vice versa.

As the included studies were performed at independent facilities using different radiation schedules, random effects models were adopted regardless of heterogeneity. Heterogeneity was considered to be present if the p value in Cochran’s Q test [41] was < 0.1 and the I^2^ value was > 50%. Sensitivity analyses were conducted by excluding 1 study at a time and reanalyzing the remaining to test whether the results had changed substantially by any individual study. Publication biases were assessed using visual inspection of funnel plots and quantitatively assessed using Egger’s test for the intercept [21]. A p value of < 0.05 was considered statistically significant. All the statistical analyses were conducted using Stata version 15.1 (Stata Corporation, College Station, TX, USA), or R (R Core Team, 2019).

## Results

### Study characteristics

**Fig. 1:**
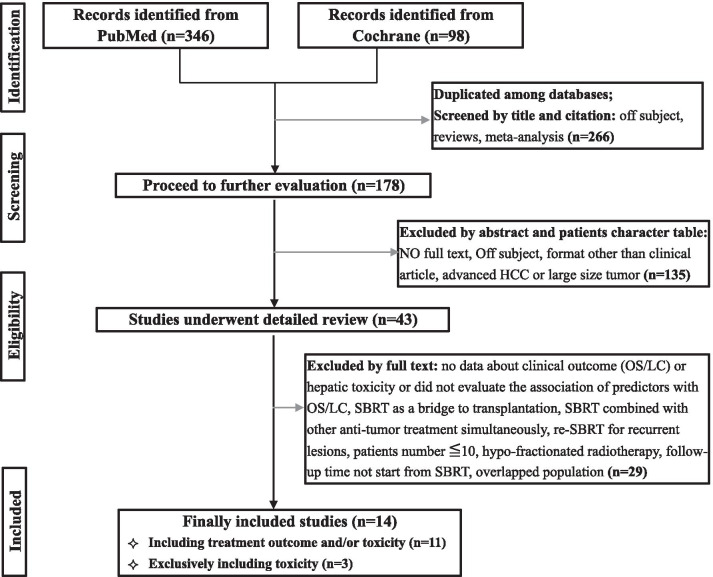
Flowchart of study inclusion

An initial search of the two databases identified 444 studies. After exclusion of unqualified studies, finally, 14 studies [8–12, 16–19, 22–26] consisting of 1238 patients, fully meeting the inclusion criteria, were eligible for the present meta-analysis. The process of study recruitment was shown in Fig. 1.

The majority of the included studies (12/14, 85.7%) featured a retrospective design. Application of the NOS revealed all of the included studies to be of medium to high quality (Table 1). In 4 of 14 studies (28.6%), only patients with small, single lesion were included, remaining studies included both single and multiple lesions (≤ 3). And most studies (11/14) included pretreated patients, with left 3 studies involving patients sole initially treated with SBRT. The age range of HCC patients was 30–90 years and 73.9% of patients were male. All the patients were ECOG performance status 0–2 and Child–Pugh class A–B respectively. In 3 studies, patients with vascular invasion were included, while most others were not containing such patients and 1 study did not mention it. We summarized studies whose median tumor sizes were available [9, 10, 12, 17, 19, 22–26] to get their overall median tumor size. It was 2.3 cm (range 0.7–6.0). Total dose of SBRT and fractionation schemes were found in most studies though quite heterogeneous. A biologically effective dose (BED) for the prescription dose was calculated using the standard linear-quadratic model (BED = D* (1 + d/α/β), D means total dose, d means dose per fraction). The calculation used the α/β ratio of 10, to consider tumor radiobiology rather than that of normal tissues. It is important to optimize values of α/β in future studies, but currently it has still been common to use α/β = 3 Gy for some normal tissues and α/β = 10 Gy for some fast turnover tissues, such as tumors whose cell survival curves do not exhibit a pronounced shoulder [27]. The median value of all available median BED_10_ estimates was 100 Gy (range 59.5–180.0 Gy). Detailed information about the included studies were shown in Tables 2 and 3.

**Table 1:** Assessment of study quality by Newcastle–Ottawa scale

Study	Selection				Comparability		Ascertainment of exposure/outcome			Score
	1	2	3	4	5	6	7	8	9	
Sun et al. [10]	√	√	√	√	×	√	√	√	√	8
Parikh et al. [11]	√	√	√	√	×	×	√	×	√	6
Jun et al. [22]	√	√	√	√	×	×	√	×	√	6
Jun et al. [17]	√	√	√	√	√	×	√	√	√	8
Jeong et al. [19]	√	√	√	√	×	√	√	√	√	8
Su et al. [23]	√	√	√	√	√	√	√	×	√	8
Takeda et al. [26]	√	√	√	√	×	×	√	√	√	7
Scorsetti et al. [8]	√	√	√	√	√	√	√	×	√	8
Kimura et al. [24]	√	√	√	√	√	√	√	√	√	9
Shiozawa et al. [12]	√	√	√	√	×	×	√	×	√	6
Naoko Sanuki et al. [25]	√	√	√	√	×	×	√	√	√	7
Yoon et al. [9]	√	√	√	√	×	×	√	√	√	7
Jung et al. [18]	√	√	√	√	√	√	√	√	√	9
Kwon et al. [16]	√	√	√	√	√	√	√	√	√	9

For cohort studies: 1, truly representative of exposed cohort; 2, non-exposed cohort drawn from the same community; 3, ascertainment of exposure; 4, outcome of interest not present at start; 5, cohorts comparable on basis of tumor stage (BCLC/UICC), or tumor size, or Child–Pugh class/score according to different research endpoint (tumor stage was used if studies mainly on treatment outcome OS, or tumor size was used if studies mainly on treatment outcome LC, or CP class/score was used if studies only about toxicity); 6, cohorts comparable on other factors (for example, we use liver Dmean for SBRT toxicity, CP class/score for OS, radiation dose for LC); 7, quality of outcome assessment; 8, follow-up long enough for outcome to occur (median/mean FU ≥ 12 months for adverse effect, and ≥ 24 months for treatment outcome OS/LC); and 9, complete accounting for cohort

**Table 2:** General and clinical information of the included studies

Authors	Year	Time of study, design	Country	No. of patients (lesions)	Inclusion of pretreated patients	Sex (M/F)	Median age (range)	CP-A%	ECOG	Viral etiology (HBV + HCV %)	Inclusion of vascular invasion patients	Median/mean longest diameter (range, cm)
Sun et al. [10]	2019	2011–2014, R	China	108 (108)	No	80/28	54 (37–77)	100%	0–1	89.8%	No	2.3 (0.7–4.9)
Parikh et al. [11]	2018	2004–2011, R	USA	32 (NA)	No	20/12	66 (72,71) IQR	NR	NR	NR	No	≤ 5.0
Jun et al. [22]	2018	2011–2016, R	Korea	85 (125)	Yes	65/20	Mean 62.6 ± 10	83.5%	NR	68.2%	No	2.23 ± 1.17
Jeong et al. [19]	2018	2012–2013, R	Korea	119 (139)	Yes	97/22	60 (36–90)	90.8%	0–2	87.4%	No	1.7 (≤ 6.0)
Su et al. [23]	2016	2009–2015, R	China	132 (175)	Yes	110/22	58 (30–88)	86.4%	NR	90.2% (HBV only)	No	3.0 (1.1–5.0)
Scorsetti et al. [8]	2015	2010–2014, P	Italy	43 (63)	Yes	31/12	Mean 72 (46–87)	53.5%	0–2	69.8%	Yes	(≤ 6.0)
Kimura et al. [24]	2015	2008–2013, R	Japan	65 (74)	Yes	44–21	73 (49–90)	86.2%	0–1	90.8%	No	1.6 (0.5–5.4)
Shiozawa et al. [12]	2015	2011–2014, R	Japan	35 (35)	No	24/11	Mean 75.1 (55–89)	80.0%	0–2	77.1%	NR	2.86 ± 1.15 (1.2–5.0)
Sanuki et al. [25]	2014	2005–2012, R	Japan	185 (185)	Yes	119/66	74 (40–89)	85.4%	NR	82.2%	Yes	2.4(0.8–5.0)
Yoon et al. [9]	2013	2007–2009, R	Korea	93 (103)	Yes	75/18	61 (42–86)	74.2%	NR	87.1%	No	2.0(≤ 6.0)
Kwon et al. [16]	2010	2004–2007, R	Korea	42 (NA)	Yes	32/10	Mean 60.1 ± 10.9	90.5%	0–1	85.7%	No	(≤ 6.0)^a^
Jun et al. [17]	2018	2011–2015, R	Korea	117 (136)	Yes	86/31	63 (38–85)	76.1%	NR	65.0%	No	2.1 (1.0–4.0)
Takeda et al. [26]	2016	2007–2012, P	Japan	90 (90)	Yes	58/32	73 (48–85)	91.1%	0–2	88.9%	Yes	2.3 (1.0–4.0)
Jung et al. [18]	2013	2007–2009, R	Korea	92 (NA)	Yes	74/18	61 (42–86)	73.9%	0–2	75.0% (HBV only)	No	(≤ 6.0)

*R* retrospective, *P* prospective, *NR* not reported, *IQR* interquartile range;

^a^Diameter is roughly calculated from volume, assuming tumor is spherical

**Table 3:** Treatment information and outcomes of the included studies

Authors	Year	SBRT dose, fractionation scheme	BED_10,_ Gy median (range)	1-year OS	3-year OS	1-year LC	3-year LC	Grade ≧ 3 hepatic complication (%)	RILD (%)	Follow-up, months
Sun et al. [10]	2019	48–54 Gy/5-8F	100 (76.8–102.6)	96.3%	80.6%	98.1%	95.1%	0/108(0%)	8/108(7.4%)	42 (6–77)
Parikh et al. [11]	2018	NR	NR	78.1%	–	–	–	–	–	16.2 (IQR: 13.4–26.9)
Jun et al. [22]	2018	40–60 Gy/3-5F	(72–180)	–	–	91.1%	89.9%	–	–	NR
Jeong et al. [19]	2018	30–60 Gy/3-4F	104.06 (60–180)	99.2%	83.8%	98.5%	97.0%	4/119 (3.4%)	10/119(8.4%)	25.8 (3.2–36.8)
Su et al. [23]	2016	42–46 Gy/3–5F 28–30 Gy/1F	(77.3–162.5)	94.1%	73.5%	90.9%	–	6/132 (4.5%)	–	21.0 (3–76)
Scorsetti et al. [8]	2015	48–75 Gy/3F 36–60 Gy/6F	(57.6–262.5)	77.9%	–	85.8%	–	7/43 (16.3%)	–	8 (3–43)
Kimura et al. [24]	2015	48 Gy/4F	105.6	92.0%	–	100%	–	–	–	26
Shiozawa et al. [12]	2015	NR	NR	95.2%	–	97.1%	–	–	–	12.6
Sanuki et al. [25]	2014	35 or 40 Gy/5F	72 (59.5 or 72)	95.0%	70.0%	99.0%	91.0%	–	–	24 (3–80)
Yoon et al. [9]	2013	30–60 Gy/3-4F	104.06 (60–180)	86.0%	53.8%	94.8%	92.1%	–	–	25.6 (1.8–55.4)
Kwon et al. [16]	2010	30–39 Gy/3F	(60–89.7)	92.9%	58.6%	72.0%	68.0%	–	–	28.7 (8.4–49.1)
Jun et al. [17]	2018	40–60 Gy/3-5F	(72–180)	–	–	–	–	–	29/117 (24.7%)	22.5 (3–56)-after SBRT
Takeda et al. [26]	2016	35 or 40 Gy/5F	(59.5 or 72)	–	–	–	–	2/90 (2.2%)	–	41.7 (6.8–96.2)
Jung et al. [18]	2013	30–60 Gy/3-4F	(60–150)	–	–	–	–	6/92 (6.5%)	17/92 (18.4%)	25.7 (1.8–55.4)

*NR* not reported

### Treatment outcomes

#### Pooled OS/LC rate and subgroup analysis

Of 14 studies, 10 reported one-year OS rate [8–12, 16, 19, 23–25] and 6 reported three-year OS rate [9, 10, 16, 19, 23, 25]. The weighted mean values of 1-year OS, 3-year OS rates were 92.8% (range 77.9–99.2%), 71.9% (range 53.8–83.8%), respectively. One-year LC was available in 10 of 14 studies [8–10, 12, 16, 19, 22–25], ranging from 72.0 to 100% with a weighted mean of 94.6%. Six of 14 studies [9, 10, 16, 19, 22, 25] reported 3-year LC ranging from 68.0 to 97.0%, with a weighted mean of 91.3%. Treatment information and outcomes are summarized in Table 3.

**Fig. 2:**
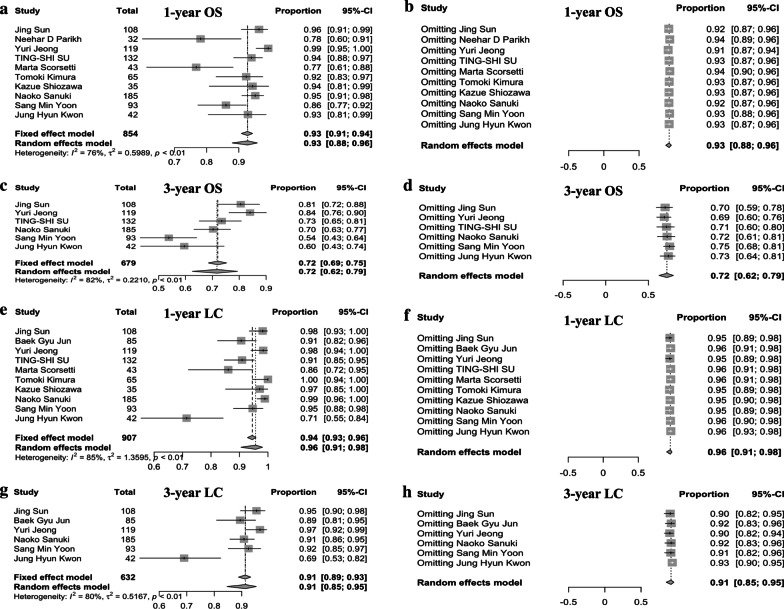
Forest plots of pooled rates of treatment outcome and sensitive analyses. **a**, **b** Pooled 1-year OS rate and related sensitive analyses; **c**, **d** pooled 3-year OS rate and related sensitive analyses; **e**, **f** pooled 1-year LC rate and related sensitive analyses; **g**, **h**, pooled 3-year LC rate and related sensitive analyses

**Fig. 3:**
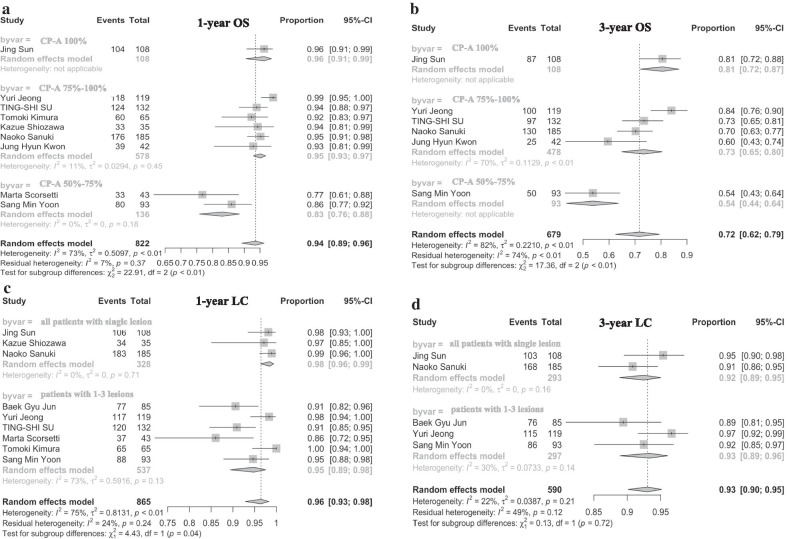
Forest plots of subgroup comparisons for treatment outcome. **a** The impact of CP-class on 1-year OS rate; **b** the impact of CP-class on 3-year OS; **c** the impact of number of lesions on 1-year LC; **d** the impact of number of lesions on 3-year LC

Pooled rates using random effects analyses of 1-year, and 3-year OS were 93.0% (95% confidence interval [CI] 88.0–96.0%), and 72.0% (95% CI 62.0–79.0%), respectively (Fig. 2a, c). Significant heterogeneities among included studies were present in the two OS rates (I^2^ > 50%, p < 0.1). Sensitive analyses were carried out by excluding 1 study at a time and reanalyzing the remaining and it showed the results had not changed substantially by any individual study (Fig. 2b, d). In subgroup comparisons, differences between subgroups categorized by CP-A percentage (stratified by 100%, 75–100%, 50–75%) were statistically significant for both 1-year OS rate (p < 0.01) and 3-year OS rate (p < 0.01), and the comparison could partly resolve statistical heterogeneity (Fig. 3a, b). For subgroup comparisons categorized by other factors, including number of lesions (single lesion or inclusion of 2–3 lesions), pretreated or not, age (median/mean age of 65), macrovascular invasion, tumor size (stratified by median/mean longest diameter of ≤ 2 cm and > 2 cm; and stratified by maximum diameter ≤ 5 cm and 5–6 cm), and radiation dose (median BED_10_ estimates of 100 Gy), no statistical differences were found among comparisons for 1-year OS and 3-year OS (Additional file 1: Supplementary Figs. 1, 2).

Pooled rates using random effects analyses of 1-year LC, and 3-year LC were 96.0% (95% CI 91.0–98.0%), and 91.0% (95% CI 85.0–95.0%), respectively. Significant heterogeneities among included studies were present in the two LC rates as well (I^2^ > 50%, p < 0.1) (Fig. 2e, g). Sensitive analyses were carried out and showed the results had not changed substantially by any individual study (Fig. 2f, h). In the subgroup comparisons regarding number of lesions (1 lesion or inclusion of 2–3 lesions), differences were statistically significant for 1-year LC rate (p = 0.04), but not for 3-year LC rate (p = 0.72) (Fig. 3c, d). It implied small HCC patients with less lesion might have better short-term local control when treated with SBRT, though in the long run, the effect was limited. For subgroup comparisons categorized by pretreatment situation, age (median/mean age of 65), CP-A percentage (stratified by 100%,75–100%,50–75%), macrovascular invasion, tumor size (stratified by median/mean longest diameter of ≤ 2 cm and > 2 cm; and stratified by maximum diameter ≤ 5 cm and 5–6 cm), and radiation dose (median BED_10_ estimates of 100 Gy), no significant differences were found among comparisons for 1-year LC or 3-year LC (Additional file 1: Supplementary Fig. 3, 4). In regarding to radiation dose, total dose (BED_10_ ≤ 100 Gy vs.  > 100 Gy) was not a significant predictor for LC in this study, but the interpretation should be very cautious because only two studies seemed to be appropriate for subgroup analysis in lower total dose (Additional file 1: Supplementary Fig. 3B) and Jing Sun et al. [10] was borderline (The median total dose was 100 Gy). When we re-grouped the studies into two groups with a BED_10_ of 100 Gy as the cutoff (BED_10_ < 100 Gy vs.  ≥ 100 Gy) and re-run the data. The results were slightly different. It showed total dose (BED_10_ < 100 Gy vs.  ≥ 100 Gy) was a significant predictor for 3-year LC (Additional file 1: Supplementary Fig. 5D) though not for 1-year LC or 1-year OS or 3-year OS (Additional file 1: Supplementary Fig. 5A–C).

#### Predictors for OS by using pooled HR meta-analysis

In order to further explore clinical predictors of OS and LC in small liver-confined HCC patients treated with SBRT, we applied the second method by combining HR and 95% CIs aim to identify parameters which can help clinicians make the therapeutic plan, determine stratification factors for future studies in this subset of patients.

We screened 9 of 14 studies which involved various prognostic factors for treatment outcome (OS or LC) [8–10, 16, 17, 19, 23–25]. As there were very limited studies on LC rate, or some studies could not supply data of HR and 95% CIs, or there were less than 3 studies for certain factor, we excluded all such unqualified studies. Finally, only 5 studies [10, 16, 17, 19, 23] concerning 4 predictors (CP class, tumor size, age, and sex) of OS were included for further analysis.

**Fig. 4:**
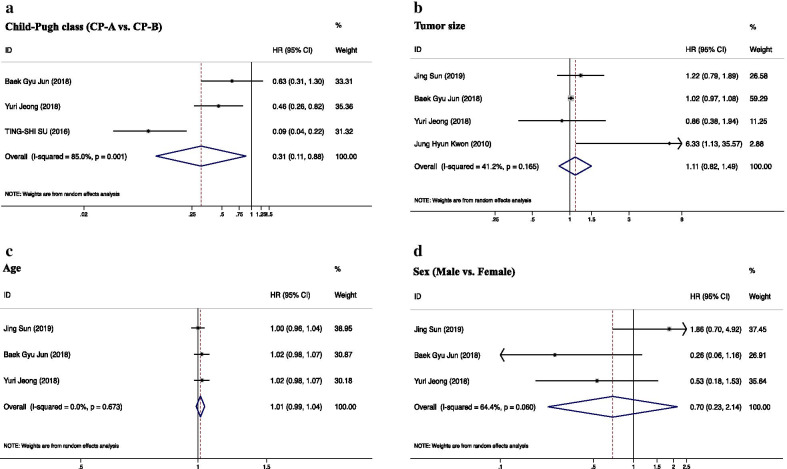
Forest plots of clinical parameters predicting OS in small HCC using SBRT. **a** The impact of Child–Pugh class on OS; **b** the impact of tumor size (including diameter and volume) on OS; **c** the impact of age on OS; **d** the impact of sex on OS

Association between CP class (A vs. B) and OS was presented in Fig. 4a. The pooled HR using random effects analysis was 0.31 with range from 0.11 to 0.88, which indicated CP-A was significantly predictive of better OS (p = 0.001). It is highly consistent with previous subgroup comparison results categorized by CP-A percentage (Fig. 3a, b). Tumor size, age, and sex were not prognostic predictors of OS with the pooled HR of 1.11 (95% CI 0.82–1.49, P = 0.165), 1.01 (95% CI 0.99–1.04, P = 0.673), and 0.70 (95% CI 0.23–2.14, P = 0.060), respectively (Fig. 4b, d).

#### Hepatic complications

**Fig. 5:**
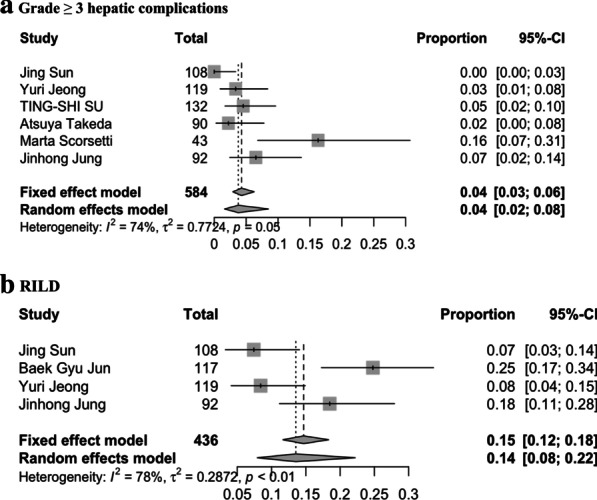
Forest plots of pooled rates of hepatic complications in small HCC using SBRT. **a** Pooled rate of grade ≥ 3 hepatic complications; **b** pooled rate of RILD

The grade ≥ 3 hepatic complications were available in 6 of 14 studies [8, 10, 18, 19, 23, 26], while the results of RILD were available in only 4 of 14 studies [10, 17–19]. The weighted mean values of the above mentioned two indexes were 4.3% (95% CI 0–16.3%) and 14.7% (95% CI 7.4–24.7%), respectively. And the pooled rates of the two indexes using random effects meta-analysis were 4.0% (95% CI 2.0–8.0%) and 15.0% (95% CI 8.0–22.0%), respectively (Fig. 5a, b). As the limited number of included studies, we did not do subgroup comparison or pooled HR meta-analysis to explore the impact factors.

#### Publication bias analysis

**Fig. 6:**
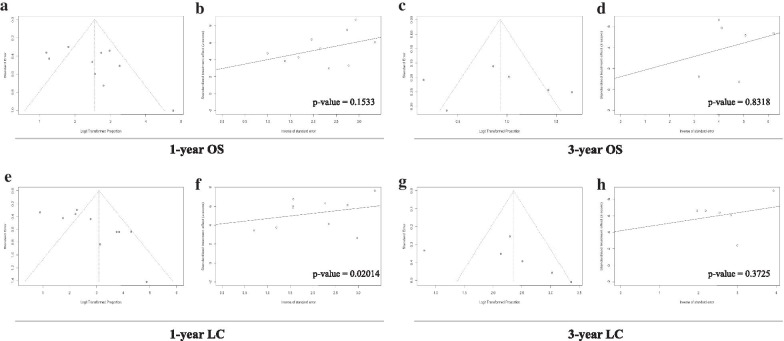
Publication biases of included studies showing in funnel plots and Egger’s test

Both visual inspections of funnel plots and Egger’s test were carried out to test the publication biases quantitatively. Publication biases were identified from 1-year LC rate studies (p = 0.02014) while not from 3-year LC rate, 1-year OS rate, and 3-year OS rate studies (p > 0.05) (Fig. 6a–h).

## Discussion

There we performed a meta-analysis of 14 studies encompassing 1238 patients who were treated with SBRT for small liver-confined HCC (≤ 3 lesions with maximum single tumor diameter ≤ 6 cm, no lymph node or extrahepatic metastasis). About the definition of small HCC, there is still no consensus in literature. In general, small sized HCC defined as ≤ 2 cm in EASL guideline, and fewer than three lesions with cumulative diameter ≤ 9 cm or single lesion up to 5 cm in MILAN criteria [13, 28]. But we still find some publications use the same definition as we do (longest diameter ≤ 6 cm and ≤ 3 lesions). Expansion of the inclusion criteria in our study to 6 cm for longest diameter per lesion as SBRT candidates is due to active research field in some institutes from different countries especially in Korea [9, 18, 19], and results from multiple retrospective studies have proved its safety and good survival for SBRT among such tumor size [8, 16, 18, 19, 24, 29]. In order to collect the actual clinical data, and distinguish it from the large lesion, we use “small” confined HCC there. Based on the criteria, our pooled results of 1-year OS, 3-year OS, 1-year LC, and 3-year LC rates were 93.0% (95% CI 88.0–96.0%), 72.0% (95% CI 62.0–79.0%), 96.0% (95% CI 91.0–98.0%), and 91.0% (95% CI 85.0–95.0%), respectively. And pooled rates of grade ≥ 3 hepatic complications and RILD were 4.0% (95% CI 2.0–8.0%) and 14.7% (95% CI 7.4–24.7%), respectively. Despite inherent heterogeneity among observational studies, these results showed that SBRT is a feasible and safe local ablative modality with potent tumor control ability and survival benefit.

Small non-metastatic HCC is associated with good prognosis due to early or intermediate stage, and it is considered candidate for definitive treatment. According to European Association for the Study of the Liver (EASL) Clinical Practice Guidelines [30] and National Comprehensive Cancer Network (NCCN) Clinical Practice Guidelines in patients with early tumors, liver transplantation or tumor resection is preferable while ablation, arterially directed therapies or radiation therapy are considered an optional local treatment. In recommendations, SBRT is mostly considered as an alternative first-line therapy to the ablation/embolization techniques when these therapies have failed or are contraindicated, or just as a second- or multi-line salvage or palliative treatment after disease progression or recurrence if there is sufficient uninvolved liver. Here the rationale to perform this review and meta-analysis is based on the above rising incidence of SBRT treatment for small liver-confined HCC, no matter patients were initial or pretreated. Given that there are several treatment options for small HCC, accurate knowledge regarding patient survival after SBRT and the determination of significant factors that impact treatment outcome after SBRT are important for optimal treatment planning.

Liver transplantation was mostly restricted to a subgroup of patients meeting the Milan selection criteria (single tumors ≤ 5 cm in diameter or no more than three nodules ≤ 3 cm in diameter in patients with multiple tumors). 1-year OS rate after liver transplantation ranged from 84 to 90% and 3-year OS rate was about 70% [28, 31, 32]. Liver resection was associated with a 1-year, 3-year, and 5-year survival rate of 85–93.3%, 62–76%, and 51–70% respectively, for selected patients with preserved liver function and early-stage HCC [28, 32–35]. In an ablative procedure, RFA is the most popular treatment option with previously published reports supporting its efficacy among early HCC patients [29, 36]. Results of some long-term study [36] showed that RFA as a first-line treatment for up to three HCCs with a maximum diameter of 5 cm, whose cumulative incidence of local control rate was 85.5% at 5 years, and overall 5-year survival rate was 67.9%, respectively. And RFA alone for the treatment of smaller (≤ 3 cm) HCCs, the treatment outcome was more optimal with 1-year, 3-year LC rate of 88.6%, 85.6%, and 1-year, 3-year OS rate of 100%, 84.5%, respectively [29]. In our group of patients (≤ 3 lesions with longest diameter ≤ 6 cm), the pooled 1-year and 3-year OS rates of SBRT were about 93.0% and 72.0%, respectively, while pooled 1-year and 3-year LC rates were about 96.0% and 91.0%, respectively. SBRT seemed to be non-inferior to surgery, and even had better LC rate than RFA although the included SBRT patients had worse prognostic criteria (larger tumor size including patients with 5–6 cm diameter, pretreated, or some with macrovascular invasion). Such comparisons should, however, be made with caution. Both different selection criteria for patients qualified for recommended treatment modalities and varying quality of reporting in studies included in this review, may potentially introduce bias. Anyway, due to the strict indications [3, 4] or postoperative complications of surgery, such as bleeding, wound infections, graft rejection [37], and anatomical difficulties in approaching some lesions or heat sink effect of RFA [5], SBRT is a promising local modality with good tumor control ability and survival benefit. A recently published systematic review on SBRT for early-stage HCC showed [38] mean weighted OS across studies was 90.9% and 73.4% at 1 and 3 years, respectively, and mean weighted LC rate across studies was 94% and 93% at 1 and 3 years, respectively. Their results are slightly different to our results. But it should be noted that, their study is not truly including early small-sized HCC as the loose inclusion criteria. It involved patients with longest reported “median” diameter < 5 cm, thus literally contained a part of patients with large tumors even up to 10 cm in diameter. In addition, C.H. Rim [5] conducted a meta-analysis including observational studies of SBRT for HCC with varied tumor size and stage published until April 23, 2018 encompassing 1950 patients. It showed pooled 1-, 2-, and 3-year OS rates were 72.6%, 57.8%, and 48.3%, respectively, and pooled 1-, 2-, and 3-year LC rates were 85.7%, 83.6%, and 83.9%, respectively. As far as we know, there are no systematic reviews or meta-analyses available on treatment outcome of SBRT for truly small liver-confined HCC to date. Our results shed a light on it.

Determination of predictive factors that impact treatment outcome after SBRT is also crucial for optimal treatment scheme. Generally, for meta-analysis there are two methods to explore potential factors. One is subgroup comparison stratified by the certain factor, though the original purpose for such analysis is to figure out the heterogeneity. The other one is using the pooled HR and 95% CIs when there were at least two studies focusing on the same factor. Comparatively, the former is much easier, while the latter is more stringent which requires the included studies to provide HR and 95% CI, and it is also a more accepted method in meta-analysis for exploring impact factors. Literally, the two methods have been attempted in our study. Both subgroup analysis and pooled HR for prognostic factors analysis strongly proved CP-A class was significantly correlated with better OS compared to CP-B class. Nevertheless, other factors evaluated in this meta-analysis including radiation dose, whether pretreated or not, tumor size, tumor number, the presence of macroscopic vascular invasion, age, and sex did not impact treatment outcome of OS among those small-sized HCC patients. To evaluate predictors of LC, because of limited studies providing data of HR and 95%CIs, only subgroup analysis was carried out. Comparison regarding number of tumor lesions showed that cohort with single lesion patients compared to cohort with 1–3 lesions patients had better 1-year LC rate (but not 3-year LC). Other factors including radiation dose, whether pretreated or not, Child–Pugh’s class, tumor size, tumor number and so forth did not impact treatment outcome of LC among those small-sized HCC patients. Of course, as mentioned earlier, we need to be very cautious about the conclusion that radiation dose is not a significant predictor for LC, because such comparisons have some limitations due to limited number of studies included. When certain borderline study [10] was cut into different group according to different criteria (BED_10_ ≤ 100 Gy vs.  > 100 Gy, or BED_10_ < 100 Gy vs.  ≥ 100 Gy), there would be different results. Whether the dose affects the outcome of treatment is still controversial. Kwon et al. [16] showed the worst LC with the lowest total dose. But Jing Sun et al. [10] showed BED_10_ did not affect LC rate, instead higher BED_10_ might improve the OS, PFS and DMFS rates. And Nitin Ohri et al. [27] also proved that among patients treated with SBRT for primary liver tumors, there was no evidence that local control is influenced by BED within the range of schedules used. For liver metastases, on the other hand, outcomes were significantly better for lesions treated with BEDs exceeding 100 Gy. It needs to be further confirmed by future large trials. In addition, we compare the prognostic factors of SBRT to that of RFA, as RFA is a mature local procedure among early small-sized HCC. Lee et al. [36] indicated 5-year OS rate was 67.9% with Child–Pugh class B as a significant predictive factors of RFA for poor survival (RR = 2.43, P = 0.011), which is similar to our results. However, it showed tumor size but not number of lesions as the only significant predictive factor of LC (RR = 2.13, P = 0.007). This is probably because the two local treatments are different in action mechanisms, and RFA is more likely subject to tumor size compared to radiotherapy. In addition, our results are inconsistent with the results of previous meta-analysis regarding the use of SBRT for HCC cases [5]. It showed subgroup comparison regarding tumor size but not CP class or number of tumor lesions had significant differences for 1- and 2-year OS rates and 1-, 2-, and 3-year LC rates. But subgroup comparisons regarding radiation dose, there were no difference for OS and LC, which is similar to our results. The reason for the differences is likely to the included patients in our studies are all small-sized cases while their study incorporated patients in various tumor size and stages. Among small HCC patients, tumor size or volume itself was not a vital factor to impact treatment outcome, and on the contrary, the liver function and number of lesions are strong impact factors.

Concerning adverse effect of SBRT, rates of grade ≥ 3 hepatic complications and RILD were mostly mild (pooled rates of 4.0% and 14.7%, respectively). However, one study [8] reported grade ≥ 3 hepatic toxicity rate was 16.3%. As limited studies supplied treatment-related toxicity and many did not distinguish between acute and late toxicities, it needs more prospective designed studies to validate. Considering the current pooled rates of complications and it might be caused by chronic liver disease itself, we support SBRT is a safe and feasible treatment for small HCC with CP-A/B class.

Of course, this study had several limitations. First and the most importantly, included studies for this meta-analysis were all observational studies which is controversial [39]. The variety of designs and populations among studies, and these differences might affect pooled estimates and inevitably brings about high heterogeneity. Though we applied random effects models for all the synthesized analysis, most results still had high heterogeneity (p < 0.1 and I^2^ > 50%). We should treat the results with caution. Further clinical trials should be prospectively investigated in large sample sizes. Secondly, we assessed a bunch of clinical factors, such as Child–Pugh class, tumor size, tumor number, and so on, but with a simplified manner by subgroup analysis. Even though we tried two methods to analyzing survival impact parameters, the included studies for more convincing pooled-HR method were in minority. Thirdly, though there were 14 studies included in this study, when they were assigned to different research endpoints, there were relatively small number of studies under each endpoint. For example, analysis of 1-year OS and LC included 10 studies, but analysis of 3-year OS and LC only included 6 studies, and pooled HR meta-analysis for OS stratified by Child–Pugh class, tumor size, age, and sex included 3, 4, 3, 3 studies, respectively. The limited number of included studies might induce great bias and lots of heterogeneity. Fourthly, as we mentioned before, there were limited data about treatment-related toxicities, which needed further studies to validate. Finally, in included studies, only a small percentage of patients were treated with SBRT as an initial treatment. Remaining patients have previously undergone other locoregional or surgical treatments. In order to reduce the influence of previous treatment, though we set an at least 1-month interval between the two therapies, there might still exist some summed effect of the previous treatment on the observed effect in a patient. However, despite the limitations, we presented analyses based on the latest and most comprehensive data and such results provide detailed information about efficacy of SBRT for small HCCs and the predictors for treatment outcome.

## Conclusion

Results of this review demonstrated SBRT was a potent local treatment for small liver-confined HCC conferring excellent OS and LC persisting up to 3 years, even though parts of included patients were pretreated or with macrovascular invasion. CP-A class was a significant predictor of optimal OS, while number of lesions might affect short term tumor control (1-year LC). Tumor size and radiation dose were not vital factors impacting treatment outcome for such small-sized HCC patients. Though the results are limited by the low quality of studies and heterogeneous groups of patients treated with SBRT, this provides a rationale for further studies applying SBRT for small HCCs as a first-line treatment or after other treatment, especially for those with single lesion and better CP class. Because of the low quality of observational studies and heterogeneous groups of patients treated with SBRT, we should treat the results with caution. But It provides a rationale for further clinical trials applying SBRT for small HCCs. We hope it can be prospectively investigated in large sample sizes.

## Supplementary Information

**Additional file 1.** Forest plots of subgroup comparisons for 1-year and 3-year OS/LC stratified by various factors, and forest plots of subgroup comparisons stratified by BED_10_ dose according to different criteria (<100Gy or ≥100Gy) for 1-year and 3-year OS/LC.

**Additional file 2.** Including 3 parts. Part 1: The detailed search query of the PubMed and Cochrane Library databases; Part 2: PICO eligibility criteria; Part 3: The detailed results of normal distribution test.

## Data Availability

All data generated or analysed during this study are included in this published article and its supplementary information files.

## Abbreviations

APPLE: The Asia-Pacific Primary Liver Cancer Expert meeting
BED: Biologically Effective Dose
CI: Confidence interval
CP: Child–Pugh
CTCAE: Common Terminology Criteria for Adverse Events
EASL: European Association for the Study of the Liver
ECOG: Eastern Cooperative Oncology Group
EORTC: The European organization for research and treatment of cancer
HCC: Hepatocellular carcinoma
LC: Local control
mRECIST: Modified RECIST
MWA: Microwave ablation
NCCN: National Comprehensive Cancer Network
NOS: The Newcastle–Ottawa Scale
NR: Not reported
OS: Overall survival
PEI: Percutaneous ethanol injection
PRIMSA: The Preferred Reporting Items for Systematic Reviews and Meta-Analysis
RECIST: The Response Evaluation Criteria in Solid Tumors
RFA: Radiofrequency ablation
RILD: Radiation induced liver disease
RTOG: The Radiation Therapy Oncology Group
SBRT: Stereotactic body radiotherapy
TACE: Transcatheter arterial chemoembolization
