# Supplementary material for: Therapeutic outcome and related predictors of stereotactic body radiotherapy for small liver-confined HCC: a systematic review and meta-analysis of observational studies
Source: Radiat Oncol. 2021 Apr 8;16:68. doi: 10.1186/s13014-021-01761-1 (PMC8034166; doi:10.1186/s13014-021-01761-1)
Supplement: Supplementary file 1 — Additional file 1. Forest plots of subgroup comparisons for 1-year and 3-year OS/LC stratified by various factors, and forest plots of subgroup comparisons stratified by BED10 dose according to different criteria (<100Gy or ≥100Gy) for 1-year and 3-year OS/LC. [file 13014_2021_1761_MOESM1_ESM.pdf]

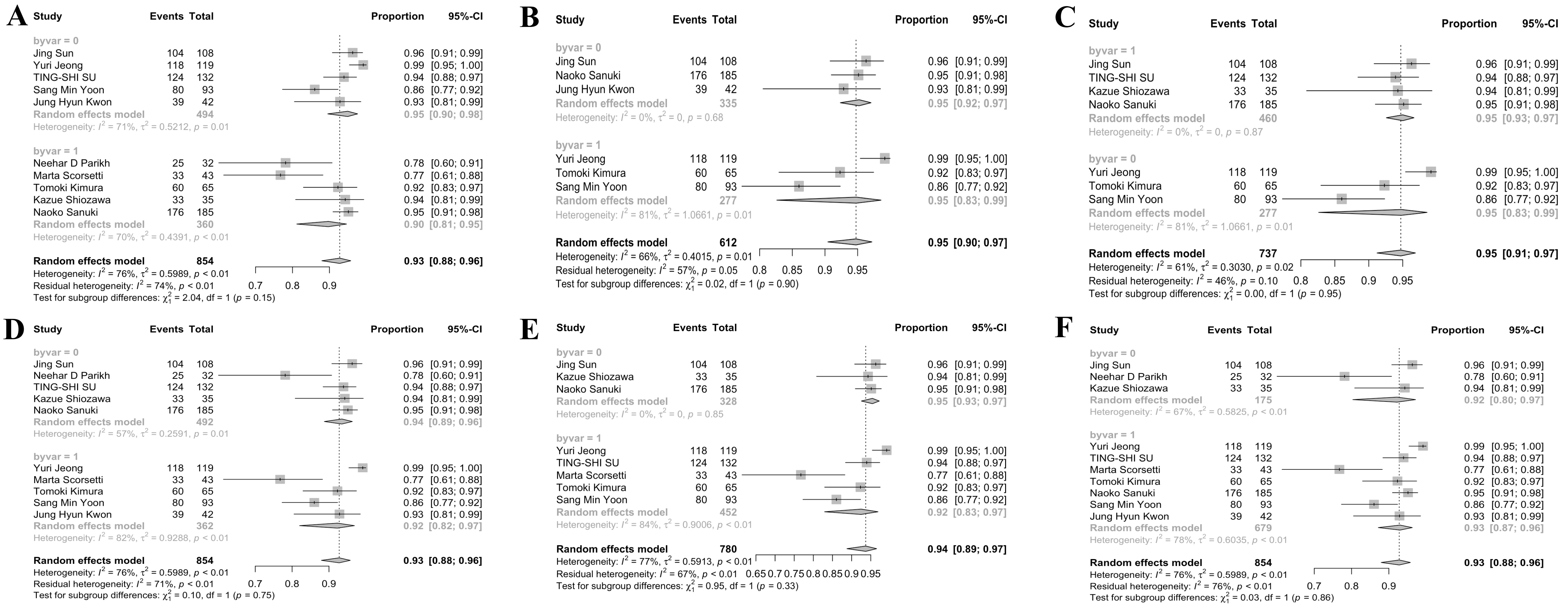

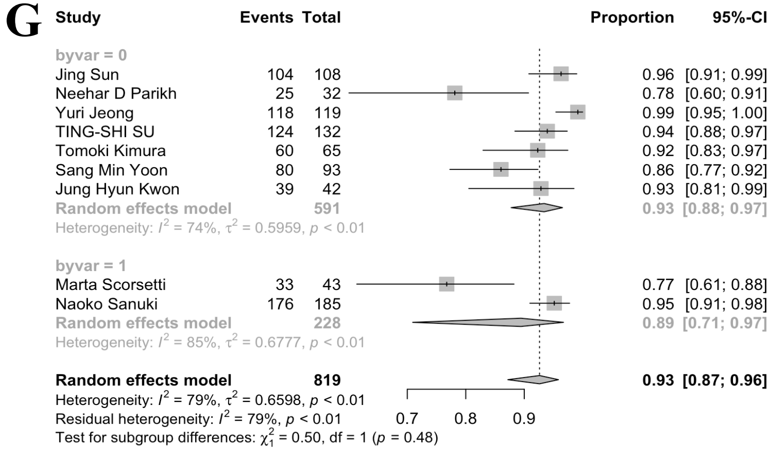

Supplementary Figure 1: **Forest plots of subgroup comparisons for 1-year OS** showing (A) age (median/mean age of <65: 0; ≥65: 1); (B) BED_{10} dose (median BED_{10} estimates of ≤100Gy: 0; >100Gy: 1); (C) tumor size (median/mean longest diameter of ≤2cm: 0; >2 cm: 1); (D) tumor size (maximum diameter ≤5cm : 0; 5-6cm : 1); (E) number of lesions (all patients with single lesion: 0; patients with ≤ 3 lesions: 1) (F) pretreatment situation (initial patients: 0; some pretreated: 1); (G) macrovascular invasion (No: 0; Yes: 1).

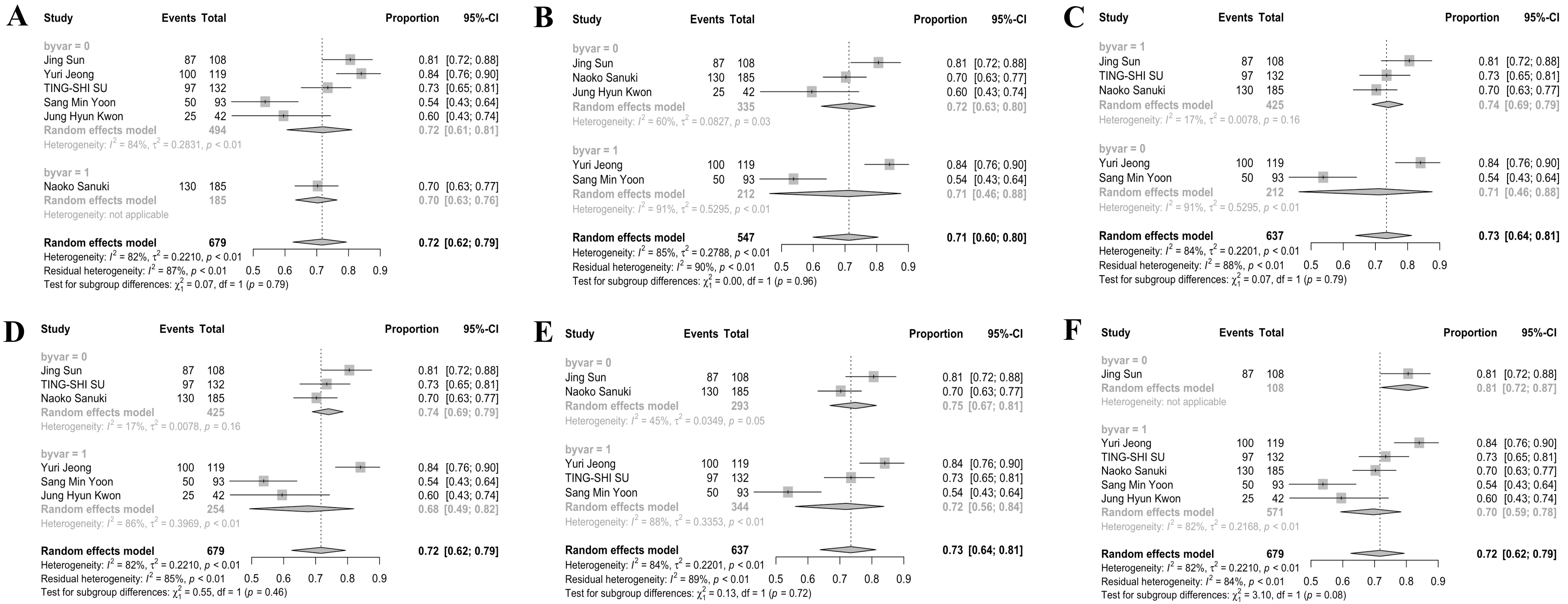

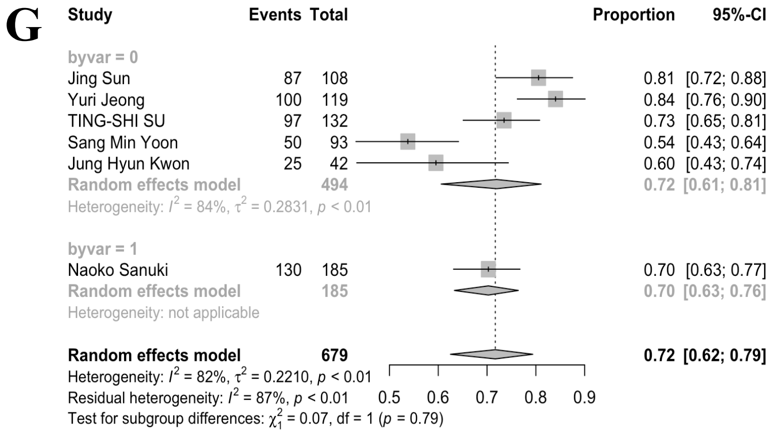

Supplementary Figure 2: Forest plots of subgroup comparisons for 3-year OS showing (A) age (median/mean age of <65: 0; ≥65: 1); (B) BED_{10} dose (median BED_{10} estimates of ≤100Gy: 0; >100Gy: 1); (C) tumor size (median/mean longest diameter of ≤2cm: 0; >2 cm: 1); (D) tumor size (maximum diameter ≤5cm : 0; 5-6cm : 1); (E) number of lesions (all patients with single lesion: 0; patients with ≤ 3 lesions: 1) (F) pretreatment situation (initial patients: 0; some pretreated: 1); (G) macrovascular invasion (No: 0; Yes: 1).

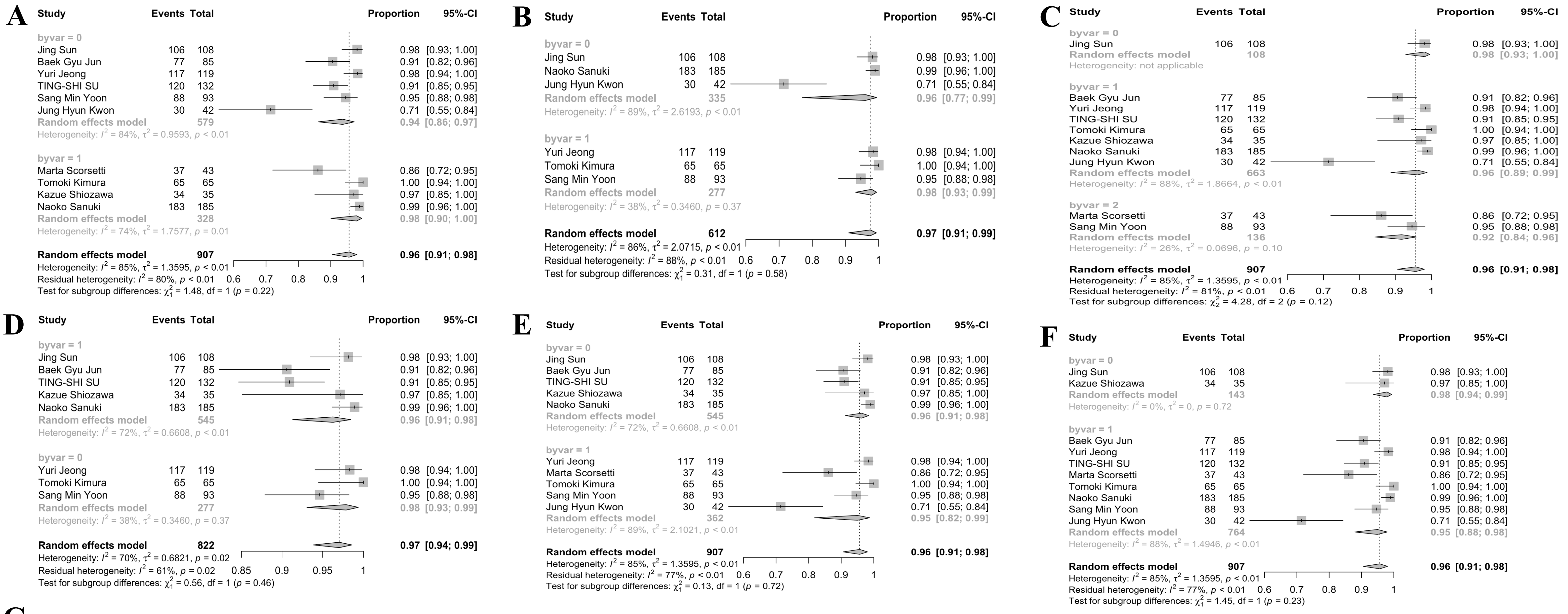

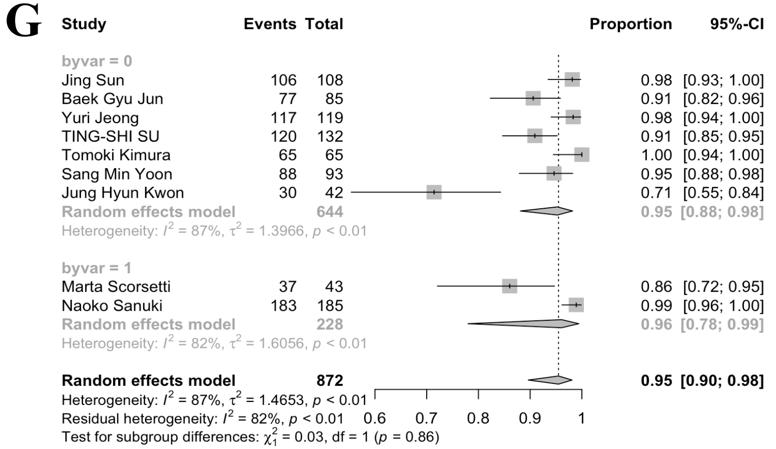

Supplementary Figure 3: **Forest plots of subgroup comparisons for 1-year LC** showing (A) age (median/mean age of <65: 0; ≥65: 1); (B) BED_{10} dose (median BED_{10} estimates of ≤100Gy: 0; >100Gy: 1); (C) CP-A percentage (100%: 0; 75-100%: 1; 50-75%: 2); (D) tumor size (median/mean longest diameter of ≤2cm: 0; >2 cm: 1); (E) tumor size (maximum diameter ≤5cm : 0; 5-6cm : 1); (F) pretreatment situation (initial patients: 0; some pretreated: 1); (G) macrovascular invasion (No: 0; Yes: 1).

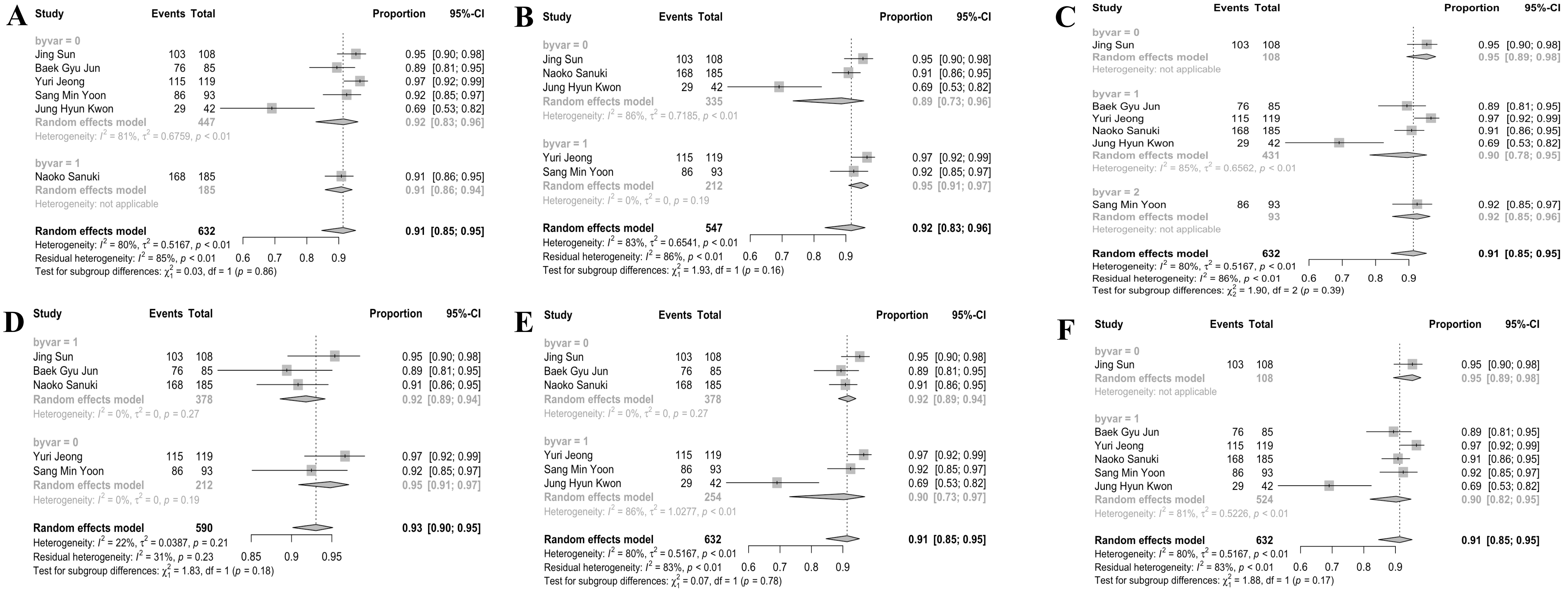

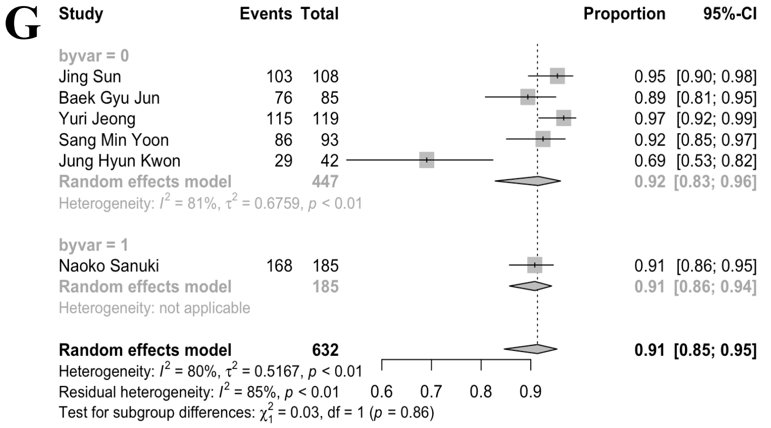

Supplementary Figure 4: **Forest plots of subgroup comparisons for 3-year LC** showing (A) age (median/mean age of <65: 0; ≥65: 1); (B) BED_{10} dose (median BED_{10} estimates of ≤100Gy: 0; >100Gy: 1); (C) CP-A percentage (100%: 0; 75-100%: 1; 50-75%: 2); (D) tumor size (median/mean longest diameter of ≤2cm: 0; >2 cm: 1); (E) tumor size (maximum diameter ≤5cm : 0; 5-6cm : 1); (F) pretreatment situation (initial patients: 0; some pretreated: 1); (G) macrovascular invasion (No: 0; Yes: 1).

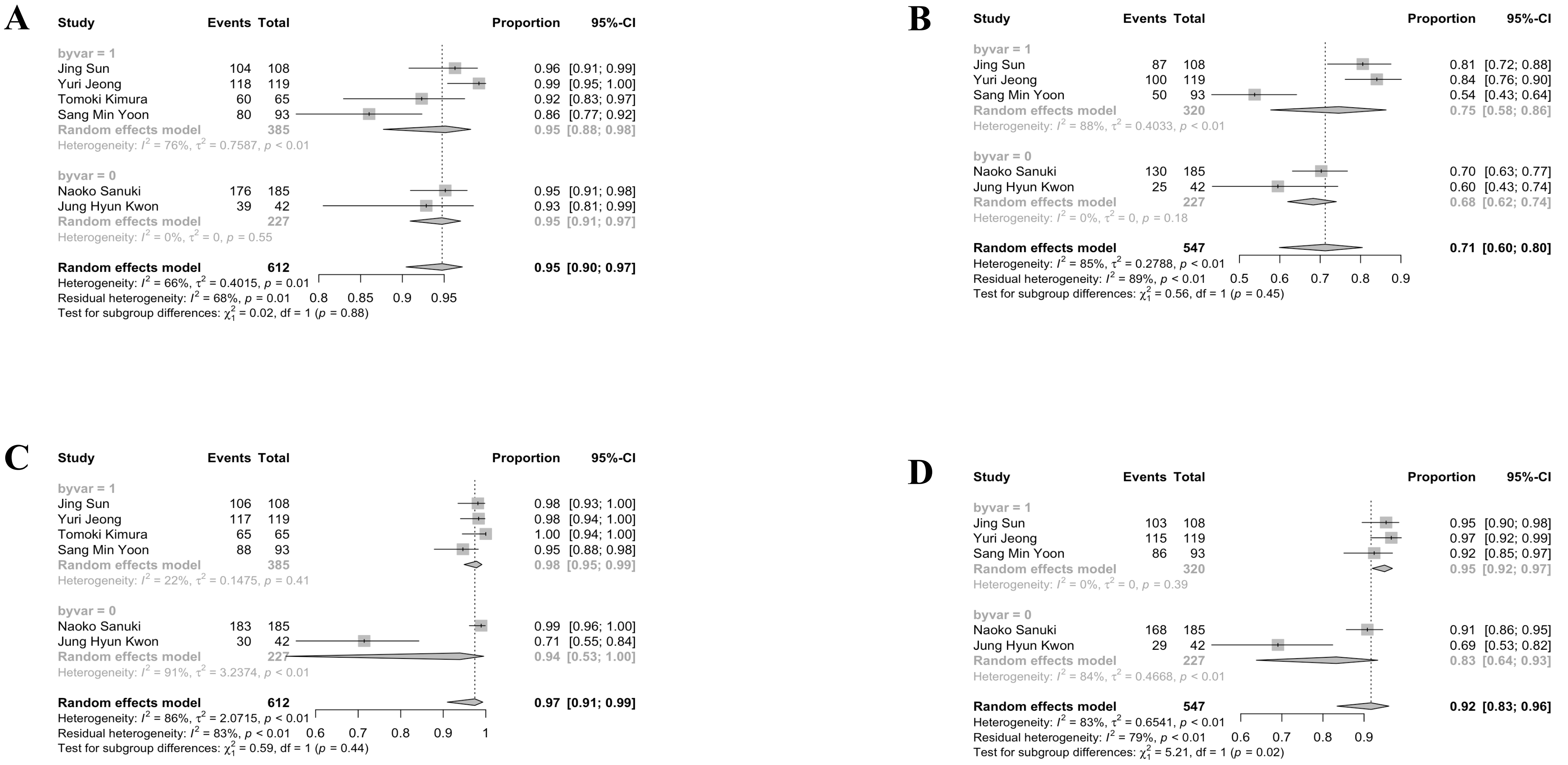

Supplementary Figure 5: Forest plots of subgroup comparisons stratified by BED10 dose (median BED10 estimates of <100Gy: 0; ≥100Gy: 1) for 1-year OS (A), 3-year OS (B), 1-year LC (C), 3-year LC (D).
