# Supplementary material for: Therapeutic outcome and related predictors of stereotactic body radiotherapy for small liver-confined HCC: a systematic review and meta-analysis of observational studies
Source: Radiat Oncol. 2021 Apr 8;16:68. doi: 10.1186/s13014-021-01761-1 (PMC8034166; doi:10.1186/s13014-021-01761-1)
Supplement: Supplementary file 2 — Additional file 2. Including 3 parts. Part 1: The detailed search query of the PubMed and Cochrane Library databases; Part 2: PICO eligibility criteria; Part 3: The detailed results of normal distribution test. [file 13014_2021_1761_MOESM2_ESM.docx]

**Part 1: Detailed search query**

1. **Pubmed database:**

Search,Query,Items found,Time
#16,"Search (English[Language]) AND ((((((((((stereotactic body radiotherapy[MeSH Terms]) OR stereotactic body radiotherap*[Title/Abstract]) OR stereotactic ablative radiotherap*[Title/Abstract]) OR Cyber*Knife[Title/Abstract]) OR Gamma*Knife[Title/Abstract]) OR SBRT[Title/Abstract]) OR SABR[Title/Abstract])) AND ((((((adenoma, hepatocellular[MeSH Terms]) OR Hepatocellular Carcinoma*[Title/Abstract]) OR hepatic malignan*[Title/Abstract]) OR liver cancer*[Title/Abstract]) OR hepatic neoplasm*[Title/Abstract]) OR liver neoplasm*[Title/Abstract])) AND ( ""2000/01/01""[PDat] : ""2020/05/01""[PDat] ) AND Humans[Mesh]) Filters: Publication date from 2000/01/01 to 2020/05/01; Humans",346,23:23:46
#15,"Search ((((((((stereotactic body radiotherapy[MeSH Terms]) OR stereotactic body radiotherap*[Title/Abstract]) OR stereotactic ablative radiotherap*[Title/Abstract]) OR Cyber*Knife[Title/Abstract]) OR Gamma*Knife[Title/Abstract]) OR SBRT[Title/Abstract]) OR SABR[Title/Abstract])) AND ((((((adenoma, hepatocellular[MeSH Terms]) OR Hepatocellular Carcinoma*[Title/Abstract]) OR hepatic malignan*[Title/Abstract]) OR liver cancer*[Title/Abstract]) OR hepatic neoplasm*[Title/Abstract]) OR liver neoplasm*[Title/Abstract]) Filters: Publication date from 2000/01/01 to 2020/05/01; Humans",373,23:22:40
#14,"Search ((((((((stereotactic body radiotherapy[MeSH Terms]) OR stereotactic body radiotherap*[Title/Abstract]) OR stereotactic ablative radiotherap*[Title/Abstract]) OR Cyber*Knife[Title/Abstract]) OR Gamma*Knife[Title/Abstract]) OR SBRT[Title/Abstract]) OR SABR[Title/Abstract])) AND ((((((adenoma, hepatocellular[MeSH Terms]) OR Hepatocellular Carcinoma*[Title/Abstract]) OR hepatic malignan*[Title/Abstract]) OR liver cancer*[Title/Abstract]) OR hepatic neoplasm*[Title/Abstract]) OR liver neoplasm*[Title/Abstract]) Filters: Publication date from 2000/01/01 to 2020/12/31; Humans",373,23:22:23
#13,"Search ((((((((stereotactic body radiotherapy[MeSH Terms]) OR stereotactic body radiotherap*[Title/Abstract]) OR stereotactic ablative radiotherap*[Title/Abstract]) OR Cyber*Knife[Title/Abstract]) OR Gamma*Knife[Title/Abstract]) OR SBRT[Title/Abstract]) OR SABR[Title/Abstract])) AND ((((((adenoma, hepatocellular[MeSH Terms]) OR Hepatocellular Carcinoma*[Title/Abstract]) OR hepatic malignan*[Title/Abstract]) OR liver cancer*[Title/Abstract]) OR hepatic neoplasm*[Title/Abstract]) OR liver neoplasm*[Title/Abstract]) Filters: Humans",375,23:21:53
#12,"Search ((((((((stereotactic body radiotherapy[MeSH Terms]) OR stereotactic body radiotherap*[Title/Abstract]) OR stereotactic ablative radiotherap*[Title/Abstract]) OR Cyber*Knife[Title/Abstract]) OR Gamma*Knife[Title/Abstract]) OR SBRT[Title/Abstract]) OR SABR[Title/Abstract])) AND ((((((adenoma, hepatocellular[MeSH Terms]) OR Hepatocellular Carcinoma*[Title/Abstract]) OR hepatic malignan*[Title/Abstract]) OR liver cancer*[Title/Abstract]) OR hepatic neoplasm*[Title/Abstract]) OR liver neoplasm*[Title/Abstract])",520,23:18:56
#11,"Search ((((((stereotactic body radiotherapy[MeSH Terms]) OR stereotactic body radiotherap*[Title/Abstract]) OR stereotactic ablative radiotherap*[Title/Abstract]) OR Cyber*Knife[Title/Abstract]) OR Gamma*Knife[Title/Abstract]) OR SBRT[Title/Abstract]) OR SABR[Title/Abstract]",18830,23:16:53
#10,"Search (((((adenoma, hepatocellular[MeSH Terms]) OR Hepatocellular Carcinoma*[Title/Abstract]) OR hepatic malignan*[Title/Abstract]) OR liver cancer*[Title/Abstract]) OR hepatic neoplasm*[Title/Abstract]) OR liver neoplasm*[Title/Abstract]",106618,23:09:40


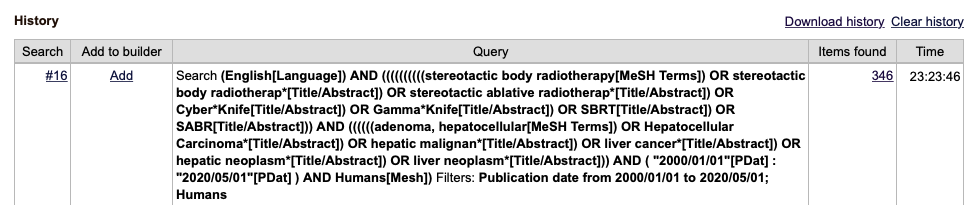


1. **Cochrane database**

ID        Search

#1        ("hepatocellular carcinoma*"):ti,ab,kw OR ("liver cancer*"):ti,ab,kw OR ("HCC"):ti,ab,kw OR ("hepatic cancer*"):ti,ab,kw OR ("liver neoplasm*"):ti,ab,kw (Word variations have been searched)

#2        MeSH descriptor: [Carcinoma, Hepatocellular] explode all trees

#3        #1 OR #2

#4        MeSH descriptor: [Radiosurgery] explode all trees

#5        ("stereotactic ablation"):ti,ab,kw OR ("stereotactic body"):ti,ab,kw OR ("Cyber Knife"):ti,ab,kw OR ("Gamma Knife"):ti,ab,kw OR (SBRT):ti,ab,kw

#6        (SABR):ti,ab,kw

#7        #4 OR #5 OR #6

#8        #3 AND #7

**Part 2: PICO eligibility criteria**

| PICO | Description |
| --- | --- |
| Patients | Patients with small liver-confined HCC (maximum single tumor diameter≦6cm or tumor volume ≦100cc), 1-3 lesions, no lymph node or extrahepatic metastasis |
| Intervention | SBRT for pretreated or initial small-sized HCC |
| Comparisons | no intervention OR TACE OR RFA OR others |
| Outcome | Local control, overall survival, predictors for LC or OS, toxicity |

**Part 3: Normal distribution test**

## L1_1_OS L1_3_OS L2_1_LC L2_3_LC L3_grade3 L3_RILD

## PRAW 0.04695441 0.7298484 0.012742599 0.01838514 0.1499892 0.3753506

## PLN 0.02924387 0.6315932 0.005084789 0.00921608 0.6505292 0.3492400

## PLOGIT 0.41294327 0.8387841 0.977341441 0.56505490 0.7248930 0.3626802

## PAS 0.42700595 0.7288792 0.209943462 0.10194077 0.9143196 0.3760822

## PFT 0.31709071 0.9292322 0.204079657 0.15433205 0.8583161 0.3412450

** p>0.05 –> normal distribution **
